# The Structural Effect of a Composite Solid Electrolyte on Electrochemical Performance and Fire Safety

**DOI:** 10.3390/ma18071536

**Published:** 2025-03-28

**Authors:** Hwiyun Im, Dae Ung Park, Yong Jae Lee, Junseok Moon, Sanglim Lee, Tae-Min Choi, Taek Lee, Giwon Lee, Jong-Min Oh, Weon Ho Shin, Sung Gyu Pyo, Anusorn Seubsai, Hiesang Sohn

**Affiliations:** 1 Department of Chemical Engineering, Kwangwoon University, Seoul 01897, Republic of Korea; gnldbs1108@gmail.com (H.I.); 2023110710@kw.ac.kr (D.U.P.); dydwo2898@naver.com (Y.J.L.); moonjune22@naver.com (J.M.); lim58432@naver.com (S.L.); biomedtlee@gmail.com (T.L.); giwonlee@kw.ac.kr (G.L.); 2 School of Integrative Engineering, Chung-Ang University, Seoul 06974, Republic of Korea; c79411@gmail.com (T.-M.C.); sgpyo@cau.ac.kr (S.G.P.); 3 Department of Electronic Material Engineering, Kwangwoon University, Seoul 01897, Republic of Korea; jmoh@kw.ac.kr (J.-M.O.); weonho@kw.ac.kr (W.H.S.); 4 Department of Chemical Engineering, Faculty of Engineering, Kasetsart University, Bangkok 10900, Thailand; fengasn@ku.ac.th

**Keywords:** organic/inorganic composite, structural effect, 0D nanoparticle, 1D nanowire network, Al-doped LLZO, PVDF-HFP

## Abstract

In this study, we investigated the structural effect of composite solid electrolytes of Al-doped LLZO and PVDF-HFP (0D_Al-LLZO@PVDF-HFP and 1D_Al-LLZO@PVDF-HFP) on electrochemical (EC) performance and fire safety through a systematic evaluation and comparative tests. The unique structure and advantageous features of composite solid electrolytes (1D_Al-LLZO@PVDF-HFP) were highlighted by comparing controls (PVDF-HFP and 0D_Al-LLZO@PVDF-HFP) with physicochemical and electrochemical analyses and fire safety tests The structure and morphology of Al-doped LLZO/PVDF-HFP composites were analyzed with X-ray diffraction (XRD) and scanning electron microscopy (SEM), while their chemical functionalities and free ion clusters were examined with Fourier transform infrared (FT-IR) spectroscopy and Raman spectroscopy, respectively. The 1D_Al-LLZO@PVDF-HFP composite with a 1D structured Al-LLZO filler network in the PVDF-HFP matrix could effectively regulate the crystallinity of PVDF-HFP and facilitated lithium salt dissociation, resulting in a high lithium-ion transference number and ionic conductivity. As a result, the 1D_Al-LLZO@PVDF-HFP composite electrolyte with an optimized structure and low Al-LLZO content (~5.1 wt%) exhibited enhanced ionic conductivity (σ: 1.40 × 10^−4^ S/cm) with low interfacial resistance, broadened EC stability (voltage window: 4.75 V vs. Li/Li^+^), and a high lithium-ion transference number (0.75) superior to that of 0D_Al-LLZO@PVDF-HFP. In electrochemical characterizations, the 1D_Al-LLZO@PVDF-HFP-based EC cell demonstrated enhanced performance in a lithium symmetric cell (>2000 h) and full cell (LiFePO_4_|electrolyte|Li) of a reversible capacity of 102.7 mAh/g at 2C with a capacity retention of 85.7% over 200 cycles, better than that of a 0D_ Al-LLZO@PVDF-HFP-based EC cell. In flammability tests, Al-LLZO@PVDF-HFP demonstrated enhanced fire safety (nonflammability) compared with that of a PVDF-HFP-based electrolyte regardless of the composite structure, suggesting the importance of inorganic filler rather than their structural morphology in the composite.

## 1. Introduction

Lithium-ion batteries (LIB), as electrochemical (EC) energy conversion/storage devices, have been used in portable electronic gadgets, electric vehicles (EVs), and energy storage systems (ESSs) due to their relatively high energy density and cycle stability [1,2,3,4]. However, the current state-of-the-art LIB is not sufficient to satisfy the performance of advanced EVs with elongated mileage and safety, requiring the development of batteries with increased energy density and improved safety [5,6,7,8]. To achieve this goal, currently, there has been various research actively performed for the development of next-generation LIB materials [9,10,11,12,13,14].

Among various materials, lithium metal has attracted significant attention as an anode material for next-generation LIBs due to its high theoretical capacity (3860 mAh/g) and low EC potential (−3.04 vs. SHE) [15]. However, the practical application of lithium metal in batteries has been hindered by its unstable EC performance caused by lithium dendrite growth and side reactions between the electrolyte and the lithium anode [16,17,18,19,20]. In addition, adopting lithium metal as an anode can debilitate battery safety due to its high reactivity and chemical instability [15,16,17,18,19,20]. In this context, extensive research has been conducted to enhance the performance and safety of lithium metal batteries (LMBs) by employing solid electrolytes, which can increase volumetric energy density and improve EC safety.

In this context, various studies have been conducted on solid electrolytes to enhance the performance and safety of LMBs, aiming to provide higher energy density per unit volume and EC stability for lithium metal anodes and overall–l safety in LMB [21,22,23]. The typical solid electrolytes include inorganics (sulfide and oxide) and organics (polymers), focusing on the ionic conductivity improvement and interfacial resistance reduction of the electrolytes [24,25]. For example, sulfide-based electrolytes exhibit high ionic conductivity (~10^−2^ S/cm), but they react with moisture in the air to form H_2_S [26,27]. In contrast, oxide ceramics (LLZO, LATP, LLTO, etc.) exhibit high stability in the oxidized environment with relatively reduced ionic conductivity (10^−4^~10^−3^ S/cm) but face challenges such as grain boundary resistance, poor electrode contact, and high sintering temperatures [28,29,30]. Among them, LLZO is regarded as a promising electrolyte material due to its wide voltage window (~6 V) and high transference number (~0.99), but it suffers from rigidity and interfacial resistance [31]. Polymer-based electrolytes (PVDF-HFP, PEO, etc.) offer advantages such as flexibility, excellent interfacial contact, and simple processing, but their practical application is limited by low ionic conductivity and instability with lithium metal [32,33,34]. Nevertheless, the application of conventional inorganic or organic solid electrolytes for LMBs is still limited and challenging because of their low ionic conductivity, high interfacial resistance (poor interfacial contact) with electrodes, insufficient mechanical properties, unstable chemical stability, unsatisfactory EC performance, and high cost [35].

As a solution to achieve improved ionic conductivity and reduced interfacial resistance, organic/inorganic composite-based solid electrolytes have been intensively pursued by combining low-crystalline polymer and ceramic particles with high ionic conductivity and mechanical stability [36,37,38]. Specifically, polymer–ceramic composite electrolytes such as ceramic-in-polymer (ceramic dispersed in a polymer matrix) [39] and polymer-in-ceramic [40] can provide enhanced ionic conductivity (reduced crystallinity of the polymer matrix), mechanical flexibilities, widen EC window, and improved interfacial contact [41,42]. Among them, LLZO filler-based composite electrolytes have been highlighted as a potential solution due to the unique role of LLZO nanofiller, including promoted lithium salt dissociation, enhanced ion conduction, and reduced energy barrier for lithium-ion transport [43]. Chen et al. demonstrated a PEO-based composite electrolyte by incorporating Li_7_La_3_Zr_2_O_12_ (LLZO) nanofillers, exhibiting enhanced EC stability attributed to the LLZO fillers dispersed in PEO with reduced crystallinity [44]. For instance, Yang et al. incorporated 5 wt% undoped cubic-phase LLZO nanowires into PAN-based polymer composite electrolytes as ceramic fillers to enhance the EC performance via the formation of continuous Li^+^ diffusion [45]. Song and coworkers incorporated a composite electrolyte of Ta- and Nb-doped LLZTNO in the PVDF matrix, exhibiting a good ionic conductivity of 1.05 × 10^−4^ S/cm [46]. Recently, we developed a composite electrolyte of Al-LLZO in a PVDF-HFP matrix for application in LMBs with enhanced electrochemical performance [47].

Despite extensive research on composite electrolytes, composite electrolytes have failed to be commercialized due to limited lithium-ion transport, a lack of network connectivity for ion conduction, and weak lithium dissociation capability, leading to unsatisfactory EC properties of localized transport bottlenecks, increased interfacial resistance, and restricted overall ionic conductivity [48,49,50]. It is required to systematically examine the structural influence of inorganic fillers on electrochemical performance and safety to achieve an optimized composite structure for solid electrolytes.

In this study, we systematically investigated the effect of composite structure on the electrochemical performance and fire safety of composite solid electrolytes of cubic-phase Al-doped Li_6.28_Al_0.24_La_3_Zr_2_O_12_ (Al-LLZO) incorporated in a PVDF-HFP/LiTFSI/SN matrix as a form of 0D nanoparticles and 1D nanowires (PVDF-HFP, 0D_Al-LLZO@PVDF-HFP, and 1D_Al-LLZO@PVDF-HFP) by comparing their properties and performance. Specifically, the composite electrolytes (0D_Al-LLZO@PVDF-HFP and 1D_Al-LLZO@PVDF-HFP) were systematically compared in terms of their electrochemical properties and thermal safety. Considering the important role of the nanofiller structure in lithium-ion transport and interfacial behavior, the unique structure of 1D_Al-LLZO@PVDF-HFP was highlighted by examining the impact of structural differences through physicochemical and electrochemical analyses, as well as fire safety tests.

Specifically, different from composite electrolytes containing inorganic 0D fillers randomly distributed in the polymer matrix, the unique structure of 1D LLZO nanowire network in 1D_Al-LLZO@PVDF-HFP can offer continuous/interconnected ion conduction networks and enhanced lithium salt dissociation, allowing for improved ionic conductivity, interfacial stability, and lithium transference number [51,52,53,54,55]. In addition, the continuous 1D Al-LLZO nanowire percolation network in the composite electrolyte enables efficient lithium-ion conduction through interconnected channels, improved lithium salt dissociation, and increased concentration of mobile Li^+^, resulting in reduced transport resistance, improved interfacial stability, and enhanced lithium transference number [54,55].

## 2. Experimental Section

### 2.1. Materials

Lithium nitrate (LiNO_3_, anhydrous, 99.999%, Sigma-Aldrich, Seoul, Republic of Korea), lanthanum nitrate hydrate (La(NO_3_)_3_·xH_2_O, anhydrous, 99.9%, Sigma-Aldrich, Seoul, Republic of Korea), zirconium(IV) propoxide solution (Zr(OCH_2_CH_2_CH_3_)_4_, 70 wt% in 1-propanol, Sigma-Aldrich, Seoul, Republic of Korea), aluminum nitrate nonahydrate (Al(NO_3_)_3_·9H_2_O, 99.9%, Alfa Aesar, Seoul, Republic of Korea), polyvinylpyrrolidone (PVP, M.W. 1,300,000, Sigma-Aldrich, Seoul, Republic of Korea), N,N-dimethylformamide (DMF, anhydrous, 99.8%, Alfa Aesar, Seoul, Republic of Korea), poly(vinylidene fluoride-co-hexafluoropropylene (PVDF-HFP M.W. 400,000, Sigma-Aldrich, Seoul, Republic of Korea), Bis(trifluoromethane)sulfonimide lithium salt (LiTFSI, 99.99%, Sigma-Aldrich, Seoul, Republic of Korea), and succinonitrile (C_4_H_4_N_2_, 99%, Thermo Scientific, Seoul, Republic of Korea) were purchased and used for our experiments without further purifications.

### 2.2. Synthesis of Al-LLZO(0D) and Al-LLZO(1D)

Al-LLZO(0D): Al-LLZO nanoparticles (Al-LLZO(0D)) were synthesized using a sol-gel method. A mixture of 7.7 mmol of LiNO_3_, 3 mmol of La(NO_3_)_3_·xH_2_O, 2 mmol of Zr(OCH_2_CH_2_CH_3_)_4_ (in 1-propanol), and 0.24 mmol of Al(NO_3_)_3_·9H_2_O was dissolved in a co-solvent of DMF and acetic acid (4:1 volume ratio) and stirred. The resulting solution was subjected to calcination at 450 °C for 1 h under ambient conditions, followed by heat treatment at 900 °C for 6 h. The obtained white powder was further ball-milled at 500 rpm for 4 h to produce cubic-phase Al-LLZO(0D) [56].

Al-LLZO(1D): Al-LLZO(1D) was prepared through consecutive sol-gel synthesis and electrospinning processes. To prepare the precursor for electrospinning, 8 wt% of PVP dissolved in DMF was mixed with a solution containing 7.7 mmol of LiNO_3_, 3 mmol of La(NO_3_)_3_·xH_2_O, 2 mmol of Zr(OCH_2_CH_2_CH_3_)_4_ (in 1-propanol), and 0.24 mmol of Al(NO_3_)_3_·9H_2_O in a co-solvent of DMF and acetic acid (4:1 volume ratio). Additionally, 10 mol% of the Li precursor was added to the mixture to prevent Li loss at high temperatures. Networked composite nanowires were collected on a stainless collector through an electrospinning process under a voltage of 18 kV. The Al-doped cubic structured Al-LLZO(1D) was prepared via consecutive sintering process of polymer (PVP) removal at 280 °C under ambient conditions, followed by as-designed crystalline structure formation for nanowire at 750 °C for 2 h.

### 2.3. Preparation of Composite Solid Electrolyte

0D_Al-LLZO@PVDF-HFP: The 0D_Al-LLZO@PVDF-HFP composite solid electrolyte was prepared by dispersing Al-LLZO(0D) inorganic fillers into the PVDF-HFP matrix at a ratio of 5.1 wt%. A solution of PVDF-HFP (1 g) and SN (0.25 g) dissolved in DMF (4 mL) was mixed with LiTFSI (0.6 g) and Al-LLZO (0.1 g) to form a slurry precursor at room temperature. The obtained slurry precursor was coated onto a glass substrate using a doctor blade, followed by vacuum drying at 60 °C for 12 h to obtain the solid electrolyte.

1D_Al-LLZO@PVDF-HFP: The 1D_Al-LLZO@PVDF-HFP composite solid electrolyte was prepared using PVDF-HFP, LiTFSI, SN, and Al-LLZO(1D) as precursors, with the same composition as the 0D_Al-LLZO@PVDF-HFP. The resulting slurry precursor was coated onto a glass substrate using a doctor blade, followed by vacuum drying at 60 °C for 12 h to obtain the solid electrolyte. The fabricated 1D_Al-LLZO@PVDF-HFP contained 5.1 wt% of Al-LLZO. Like the preparation of the 0D_Al-LLZO@PVDF-HFP composite, SN was added as a plasticizer to enhance the ionic conductivity of the electrolyte. To clearly distinguish the morphological differences of the fillers, all electrolytes were fabricated based on a PVDF-HFP/LiTFSI/SN matrix. The sample names and their corresponding full descriptions used in this study are summarized in Table 1.

### 2.4. Material Characterizations

The crystal structure of the material for the solid electrolyte was obtained with X-ray diffraction (XRD, Rigaku MiniFlex 600, Tokyo, Japan) operating at 40 kV and 15 mA using CuK_α_ (=0.15409 nm) in the two-theta range of 10° to 70°. The surface chemical functionality of composite was analyzed with Fourier transform infrared (FT-IR) spectroscopy (JASCO, FT-IR 4600, Tokyo, Japan) by analyzing the chemical interaction of molecules in the samples. In the FT-IR analysis, the material was analyzed under ATR mode without compositing with KBr pellets for sample preparation. Raman analysis for each electrolyte was conducted using a Raman spectrometer (Nanobase, XPER RAM, Seoul, Republic of Korea) with a 532 nm laser light source. The morphology of Al-LLZO and the composite solid electrolyte were analyzed with field-emission scanning electron microscopy (FE-SEM, Hitachi, S-4800, Tokyo, Japan) operated at 30 kV. Elemental distribution of Al-LLZO was determined using energy-dispersive X-ray spectroscopy (EDS) attached to SEM.

### 2.5. Electrochemical Characterizations

The positive electrode (cathode) was prepared using lithium iron phosphate (LFP, MTI Korea, Seoul, Republic of Korea) or lithium nickel manganese cobalt oxide (NCM811 (LiNi_0.8_Co_0.1_Mn_0.1_O_2_), MTI Korea, Seoul, Republic of Korea) as active material, carbon black (Super-P, Alfa Aesar, Seoul, Republic of Korea) as conductive agent, and poly(vinylidene fluoride (PVDF (M.W. 534,000), Sigma-Aldrich, Seoul, Republic of Korea) as binder. The as-prepared cathode materials (active material, conductive agent, and binder) were mixed and stirred to obtain a slurry coating. As-obtained slurry was coated on Al foil, followed by vacuum drying at 60 °C for 24 h, while the loading amount of the active material in the cathode was 3.4 mg/cm^2^. The resulting slurry was coated onto Al foil and vacuum dried at 60 °C for 24 h, with the loading amount of the active material in the cathode being 3.4 mg/cm^2^. For smooth interfacial contact with the solid electrolyte and flame retardation, 2 μL of fluoroethylene carbonate (FEC, Enchem Co., Ltd., Jecheon, Republic of Korea) was added. As-prepared cathodes were used for full-cell test by adopting various solid electrolytes.

The ionic conductivity of solid electrolyte was measured with SS|SS (SS: stainless steel) electrodes using blocking electrodes at both sides at various temperatures. Electrochemical impedance spectroscopy was measured using an impedance meter (Zive SP 1, WonAtech, Seoul, Republic of Korea) with alternating current frequency of 10 mV in the frequency range from 1 MHz to 0.1 Hz. It is important to note that the ionic conductivity (σ) of the solid electrolyte was calculated using Equation (1) as shown below, where *R_b_* is the bulk resistance of the solid electrolyte, and *S* is the area of the solid electrolyte [57].

(1) $$\sigma =\frac{L}{R_{b}S}$$

The activation energy (*E*_a_) of the solid electrolyte was calculated using Equation (2) at various temperatures from 30 °C to 70 °C, where *σ* is the ionic conductivity, *T* is the absolute temperature, *A* is the pre-exponential factor, and *k* is the Boltzmann constant [58].

(2) $$\sigma T=Aexp(\frac{-E_{a}}{k\times T})$$

The transference number (t_Li+_) of Li^+^ was measured with the Li|Li symmetric cell polarized at a voltage of 10 mV for 5000 s and was calculated using Vincent–Evans Equation (3), where ΔV is 10 mV, *I*_0_ and *I*_ss_ are the initial current and steady-state current measured by direct current polarization, and R_0_ and R_ss_ are the initial and steady state interfacial resistance, respectively [59].

(3) $$t_{Li+}=\frac{I_{ss}(∆V-I_{0}R_{0})}{I_{0}(∆V-I_{ss}R_{ss})}$$

The electrochemical window of the electrolyte was measured with a Li|SS cell to perform a linear sweep voltammetry (LSV) test, where LSV was measured over a range of 3 V to 6 V at a rate of 1 mV/s. The EC performance of lithium metal battery was measured with Li|LFP coin cell in the range of 2.5 V to 4.2 V at 30 °C using a battery tester (WBCS3000, WonAtech, Seoul, Republic of Korea).

### 2.6. Fire Safety Test

The safety of the as-formed solid electrolytes (PVDF-HFP, 0D_Al-LLZO@PVDF-HFP and 1D_Al-LLZO@PVDF-HFP) was evaluated with flammability test by comparing with liquid electrolyte of 1M LiPF_6_ dissolved in a solvent of ethylene carbonate (EC) and diethyl carbonate (DEC) at 1:1 volume ratio. Briefly, the flammability test for the polypropylene (PP) separator soaked in ca. 50 μL of liquid electrolyte was compared with that of solid electrolytes (PVDF-HFP, 0D_Al-LLZO@PVDF-HFP, and 1D_Al-LLZO@PVDF-HFP). The flame-retardant properties of the electrolyte were evaluated by applying a flame to electrolytes (solid and liquid electrolytes) for 1 s in air to measure their fire/flame resistance.

## 3. Results and Discussion

**Scheme 1:**
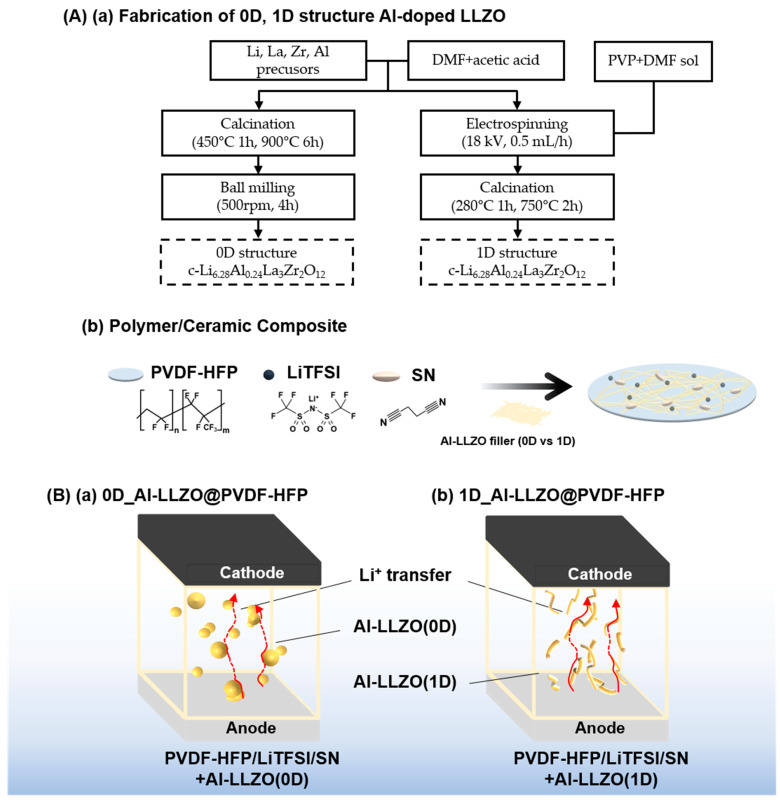
(**A**) Schematic illustration of Al-LLZO synthesis. (**a**) Preparation of the synthesis of Al-LLZO(0D) fillers via the sol-gel method, followed by Al-LLZO(1D) fillers through electrospinning. (**b**) Dispersion of Al-LLZO, LiTFSI, and SN in the PVDF-HFP polymer to form an Al-LLZO composite within the PVDF-HFP matrix. (**B**) Schematic representation of the key features of the Al-LLZO@PVDF-HFP solid electrolyte. (**a**) The 0D-structured Al-LLZO nanoparticles exhibit discontinuous lithium-ion transfer mechanism, resulting in lower ionic conductivity. (**b**) In contrast, the 1D-structured Al-LLZO nanowires provide continuous lithium-ion transport channels, contributing to enhanced ionic conductivity and electrochemical performance. The morphological differences between the fillers highlight the superior performance of Al-LLZO(1D) in the composite electrolyte.

Scheme 1 illustrates the preparation process (Scheme 1A) and composite structure (Scheme 1B) of electrolytes. In addition, the unique structure of the 1D composite electrolyte (1D_Al-LLZO@PVDF-HFP) could be highlighted by comparing the control electrolytes (PVDF-HFP and 0D_Al-LLZO@PVDF-HFP), which overcomes the limitations of conventional hybrid systems.

Scheme 1(Aa) illustrates the synthesis process of Al-LLZO with different structures (Al-LLZO (0D) and Al-LLZO (1D)) using precursors of inorganic (Li, La, Zr, and Al) salts. The 0D nanoparticle structure is synthesized via the sol-gel method, followed by a calcination process and subsequent ball milling, while the 1D nanowire structure is obtained through an electrospinning process, followed by a consecutive sintering step. Scheme 1(Ab) presents the compositing process of the electrolyte, where the sintered 0D and 1D Al-LLZO are dispersed into a PVDF-HFP matrix to fabricate the 0D or 1D Al-LLZO@PVDF-HFP (Al-LLZO/PVDF-HFP/LiTFSI/SN) solid electrolyte. It is important to note that a lithium salt (LiTFSI) and a plasticizer (succinonitrile, SN) were added to the PVDF-HFP matrix, contributing to the reduced crystallinity of PVDF-HFP, facile dissociation of LiTFSI, and improved interfacial stability with lithium metal through the molecular interaction of Li^+^ and the nitrile group (-S≡N-) [60,61,62]. Thus, the decomposed lithium salts act as a Lewis acid in the polymer, enabling diversified lithium transport channels during the electrochemical charge/discharges [63].

Scheme 1B illustrates the key features of the Al-LLZO@PVDF-HFP composite-based solid electrolyte. As shown in Scheme 1B, the 0D-structured Al-LLZO exhibits discontinuous lithium-ion transfer (Scheme 1(Ba)), whereas the 1D-structured Al-LLZO fillers provide continuous lithium-ion transport channels (Scheme 1(Bb)). As comparatively illustrated, 1D Al-LLZO nanowires in the 1D_Al-LLZO@PVDF-HFP could not only allow the formation of continuous ion transport pathways but also enhance lithium salt dissociation and interfacial compatibility through improved polymer–filler interactions. Additionally, the facilitated lithium salt dissociation by a larger surface area within the polymer matrix of Al-LLZO(1D) enhances the ion conductivity. Specifically, the major lithium-ion transport in 0D_Al-LLZO@PVDF-HFP occurs through disconnected and non-uniform ion conduction pathways among 0D nanoparticle fillers, resulting in inorganic filler aggregation and reduced EC performance with cycles. In contrast, 1D_Al-LLZO@PVDF-HFP allows the ion transport through a more straightforward pathway along 1D-structured inorganic fillers, resulting in enhanced ionic conductivity (σ: 1.40 × 10^−4^ S/cm), expanded electrochemical voltage window (4.75 V vs. Li/Li^+^), and reduced manufacturing costs of a low Al-LLZO content (~5.1 wt%). Thus, the structural difference of composite electrolytes (Al-LLZO@PVDF-HFP) can affect the EC characteristics and performance of the application for lithium metal batteries [54,55].

As compared, the unique and novel composite structure of 1D_Al-LLZO@PVDF-HFP over a control electrolyte could collectively contribute to the improved ionic conductivity, electrochemical stability, and lithium transference number, providing a significant structural and functional advantage over conventional 0D nanoparticle-based electrolytes [54,55].

**Figure 1:**
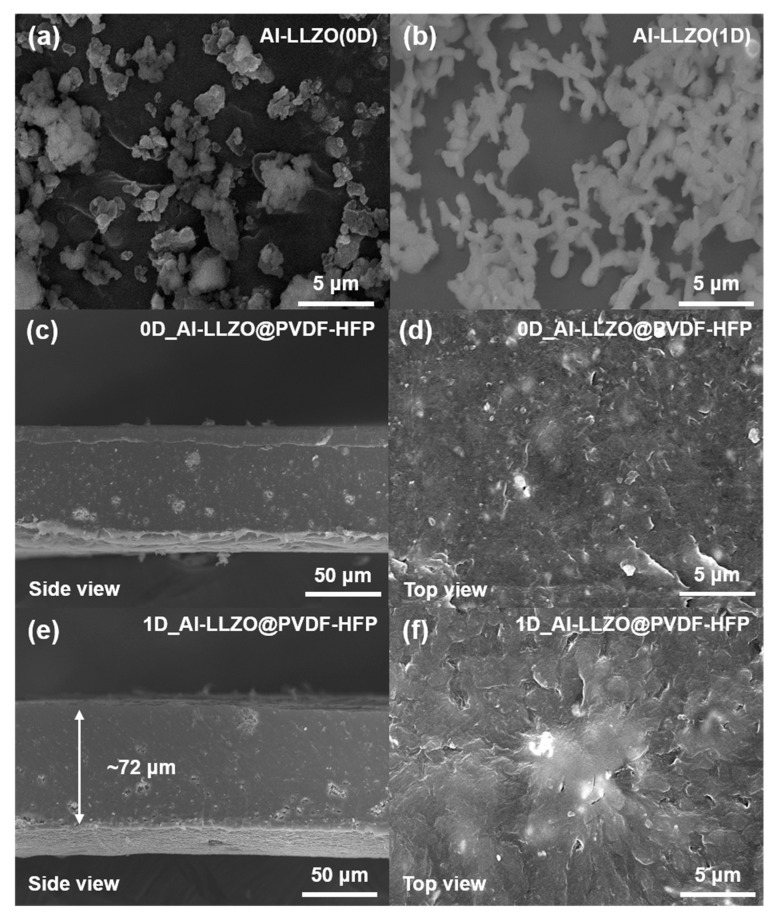
Morphology images of (**a**) Al-LLZO(0D) nanoparticles and (**b**) Al-LLZO(1D) nanowires. The SEM images of the composite electrolytes: (**c**) cross-sectional and (**d**) top-view images of 0D_Al-LLZO@PVDF-HFP and (**e**) cross-sectional and (**f**) top-view images of 1D_Al-LLZO@PVDF-HFP.

Figure 1 displays the comparative morphological analyses of the inorganic fillers (0D and 1D Al-LLZO) and composite electrolytes of 0D and 1D_Al-LLZO@PVDF-HFP obtained via scanning electron microscopy (SEM).

As shown in the image of Al-LLZO(0D) (Figure 1a), Al-LLZO(0D) exhibits an average particle diameter of approximately 800 nm after ball milling. Figure 1b shows the morphology of Al-LLZO(1D) after sintering at 750 °C, exhibiting a slightly increased diameter with a partially collapsed global structure. In comparison, for Al-LLZO(1D) (Figure S1a), uniform and bead-free PVP nanowires were observed, with an average diameter of ca. 330 nm after electrospinning, indicating the successful synthesis of 1D-structured Al-LLZO nanowire precursors. It is important to note that the major structure of Al-LLZO nanowire remains intact after sintering, forming a network within the polymer matrix (Figure S1b,c). Figure S1d presents the elemental composition (Zr, La, Al, and O) of Al-LLZO(1D) taken with SEM-EDS, revealing a homogeneous elemental distribution of Zr, La, and Al in the garnet-structured Al-LLZO. This robust structure of the Al-LLZO (1D) network provides an effective lithium-ion transport channel [45]. The obtained morphologies of PVDF-HFP (cross-sectional image (Figure S2a) and top-view image (Figure S2b)) exhibit partially dark regions and cracks in PVDF-HFP due to the formation of uneven surfaces or pores (or voids) in the electrolyte, suggesting poor interfacial contact and gaps in lithium-ion transport in the PVDF-HFP. As displayed in Figure 1c,d, top-view and cross-sectional SEM images for 0D_Al-LLZO@PVDF-HFP were obtained, clearly revealing partially dispersed Al-LLZO(0D) nanoparticles within the composite. Specifically, as seen in Figure 1c,d, Al-LLZO nanoparticle fillers are visible as partially agglomerated with fused micropores in the composite, forming a more integrated structure. As demonstrated in the plane and cross-sectional SEM images for the 1D_Al-LLZO@PVDF-HFP composite (Figure 1e,f), a continuous nanowire network embedded in the polymer matrix is clearly visible (Figure 1e), with a unified surface structure without visible pores (Figure 1f), suggesting the formation of a more densified composite structure compared with that of PVDF-HFP and 0D_Al-LLZO@PVDF-HFP (Figure 1d and Figure S2b). Such a robust composite structure of 1D_Al-LLZO@PVDF-HFP implies the contribution of nanofiller morphologies to the structural integrity and performance of composite solid electrolytes [45,46,47].

**Figure 2:**
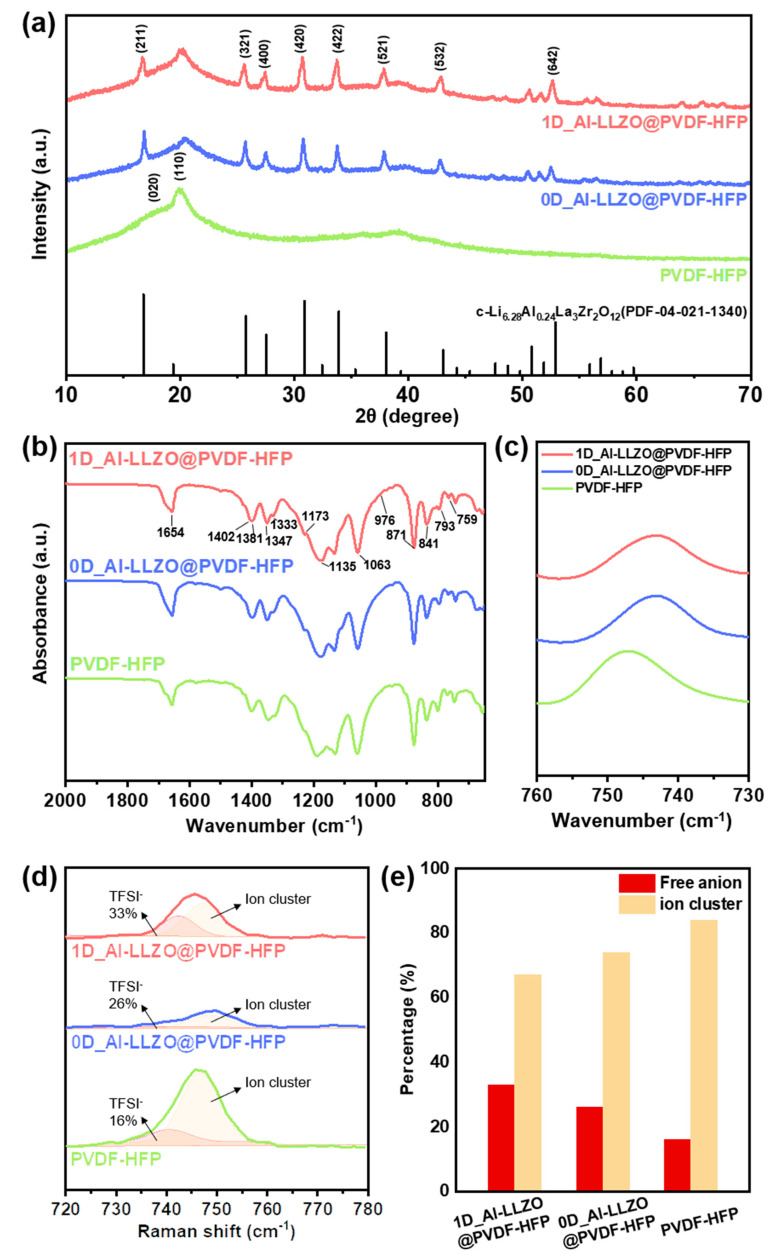
(**a**) Comparison of XRD patterns of PVDF-HFP (PVDF-HFP/LiTFSI/SN), 0D_Al-LLZO@PVDF-HFP, and 1D_Al-LLZO@PVDF-HFP. (**b**) FT-IR spectra of PVDF-HFP (PVDF-HFP/LiTFSI/SN), 0D_Al-LLZO@PVDF-HFP, and 1D_Al-LLZO@PVDF-HFP and (**c**) characteristic peaks for PVDF-HFP (PVDF-HFP/LiTFSI/SN), 0D_Al-LLZO@PVDF-HFP, and 1D_Al-LLZO@PVDF-HFP in the wavenumber range of 730 cm^−^^1^ to 760 cm^−^^1^. (**d**) Raman spectra of PVDF-HFP, 0D_Al-LLZO@PVDF-HFP, and 1D_Al-LLZO@PVDF-HFP used to estimate the relative portion of free TFSI^−^ (~740 cm^−1^) and ion clusters (~745 cm^−1^) in the composite electrolytes. (**e**) Comparison of free TFSI^−^ and ion cluster ratios in PVDF-HFP (PVDF-HFP/LiTFSI/SN), 0D_Al-LLZO@PVDF-HFP, and 1D_Al-LLZO@PVDF-HFP, demonstrating enhanced lithium salt dissociation with Al-LLZO(1D) fillers.

As displayed in Figure 2, the crystal structures were analyzed for solid electrolyte materials (0D_Al-LLZO@PVDF-HFP, 1D_Al-LLZO@PVDF-HFP, and PVDF-HFP (PVDF-HFP/LiTFSI/SN)) with XRD (Figure 2a and Figure S3), while the chemical functionality of the electrolyte materials were analyzed with FT-IR (Figure 2b,c).

Figure 2a and Figure S3a exhibit the characteristic crystalline structure of Al-LLZO in the composites (0D_Al-LLZO@PVDF-HFP and 1D_Al-LLZO@PVDF-HFP) and inorganic fillers (Al-LLZO(0D) and Al-LLZO(1D)), suggesting no formation of a secondary phase in Al-LLZO due to the sintering process at high temperature (>750 °C). As-formed impurities (Li_2_CO_3_) on Al-LLZO can induce a sluggish charge transfer at the ceramic–polymer interface due to the insulating property of Li_2_CO_3_, high interfacial resistance with polymer/Li_2_CO_3_, and biased migration of lithium ions along the boundaries of inorganic (Al-LLZO) and organic (polymer. PVDF-HFP) [64,65]. However, the as-synthesized Al-LLZO(0D) and Al-LLZO(1D) formed under high-temperature sintering can effectively regulate the crystal structure of the matrix by reducing the rigid crystalline region in the matrices and promote ion pair dissociation by adsorbing the counter anions [66]. In addition, there was no phase deformation observed for Al-LLZO(0D) and Al-LLZO(1D) dispersed in the polymer matrix (PVDF-HFP). As displayed in the crystal structures of PVDF-HFP in all electrolytes (PVDF-HFP, 0D_Al-LLZO@PVDF-HFP, and 1D_Al-LLZO@PVDF-HFP) in Figure S3b, the characteristic α and β phase peaks of PVDF-HFP (18.3° and 20.4°) were observed in all the spectra of electrolytes [67]. Moreover, the characteristic XRD peaks for SN and LiTFSI (Figure S3c) were not visible in 0D_Al-LLZO@PVDF-HFP and 1D_Al-LLZO@PVDF-HFP, indicating a good distribution/dissolution of SN and LiTFSI in the composite. It is important to note that the attenuated α phase peak of 1D_Al-LLZO@PVDF-HFP indicates the reduced crystallinity of PVDF-HFP in the composite due to the Al-LLZO addition and the polar groups (-CH_2_ and -CF_2_) in the β-phased PVDF-HFP on the Al-LLZO, leading to (1) enhanced Li^+^ transfer and (2) facilitated lithium salt (LiTFSI) dissociation [68].

As displayed in the FT-IR spectra of electrolytes (PVDF-HFP, 0D_Al-LLZO@PVDF-HFP, and 1D_Al-LLZO@PVDF-HFP) in Figure 2b,c, the peak at 793 cm^−1^ corresponds to CH_2_ rocking and CF_2_ stretching, and the peaks at 759 cm^−1^ and 976 cm^−1^ represent the α-phase of PVDF-HFP [69]. The vibration peaks at 841 cm^−1^ and 871 cm^−1^ correspond to the β-phase, while the peaks at 1063 cm^−1^, 1173 cm^−1^, 1381 cm^−1^, and 1402 cm^−1^ are attributed to the vibrations of the -C-C backbone [69], the stretching vibration of the C-F bond, the wagging vibration of -CH_2_, and the stretching of -C-F bonds, respectively [70].

Notably, as displayed in the spectra of electrolytes (Al-LLZO@PVDF-HFP and PVDF-HFP) in Figure 2b, the addition of SN and LiTFSI to PVDF-HFP reduces the α-phase peaks of PVDF-HFP, with the 759 cm^−1^ peak in 1D_Al-LLZO@PVDF-HFP decreasing more significantly than in PVDF-HFP [71]. The peak at 1135 cm^−1^ indicates interactions between Li^+^ and CF_3_ groups [72]. Additionally, two peaks around 1350 cm^−1^ (1333 cm^−1^ and 1347 cm^−1^) are linked to the C-SO_2_-N bond, while the 1347 cm^−1^ peak originates from the asymmetric SO_2_ vibration caused by LiTFSI addition [71]. As compared in the spectra for 0D_Al-LLZO@PVDF-HFP and 1D_Al-LLZO@PVDF-HFP, the 1654 cm^−1^ peak with reduced intensity is visible for 1D_Al-LLZO@PVDF-HFP, indicating decreased LiTFSI aggregation and enhanced ionization. Thus, the reduced peak intensity of LiTFSI for 1D_Al-LLZO@PVDF-HFP suggests facilitated LiTFSI dissociation due to nanowire-structured Al-LLZO [66]. Figure 2c shows a clear peak shift of 730 cm^−1^ and 760 cm^−1^ for PVDF-HFP in the electrolytes (PVDF-HFP, 0D, and 1D_Al-LLZO@PVDF-HFP), indicating the prevalent existence of free TFSI^−^ (740 cm^−1^) and contacted ion pairs (745 cm^−1^) [73]. These peak shifts at 730 cm^−1^ and 760 cm^−1^ in 0D and 1D_Al-LLZO@PVDF-HFP suggest an elongation of the S-N-S bond in TFSI^−^ ions, which can be attributed to the coordination of Li^+^ ions with TFSI^−^ anions, leading to the formation of ion pairs and clusters [74].

As displayed in Figure 2d,e, the ionic states in the composite electrolytes were examined with Raman spectroscopy. Figure 2d identifies the existence of LiTFSI in the electrolyte as free anions (TFSI^−^) and ion clusters visible in the range of 700–800 cm^−1^ [74,75]. Specifically, the peak at ~740 cm^−1^ corresponds to free TFSI^−^, while the peak at ~745 cm^−1^ represents ion clusters where the areas of these peaks were identified and calculated to estimate the relative portion of free TFSI^−^ (~740 cm^−1^) and ion clusters (Figure 2d) [76]. Figure 2e compares free TFSI^−^ and ion cluster ratios for the composite electrolytes with different structural morphologies. In PVDF-HFP without Al-LLZO fillers, a lower amount of free TFSI^−^ (16%) and a higher amount of ion clusters were estimated. However, 0D_Al-LLZO@PVDF-HFP containing Al-LLZO(0D) exhibited an increased amount of free TFSI^−^ (26%), suggesting facilitated lithium salt dissociation within the polymer matrix due to Al-LLZO(0D) filler. It is important to note that 1D_Al-LLZO@PVDF-HFP containing 1D-structured Al-LLZO showed a significantly higher portion of free TFSI^−^ (33%), indicating the more effective function of 1D Al-LLZO nanowires on lithium salt dissociation compared with that of 0D Al-LLZO. These results highlight that the continuous lithium-ion transport channels and salt dissociation induced by 1D Al-LLZO nanowires contribute to enhanced ionic conductivity and lithium-ion transport efficiency in the composite electrolyte.

Overall, as compared in Figure 2, there were no significantly different physico-chemical properties found for the composite electrolytes with different organic/inorganic composite structures (0D and 1D Al-LLZO@PVDF-HFP). Nevertheless, it is noteworthy that 1D_Al-LLZO@PVDF-HFP showed a higher portion of free TFSI^−^ (33%) compared with that of the 0D electrolyte, indicating that nanowires have a greater effect on lithium salt dissociation compared to nanoparticles.

**Figure 3:**
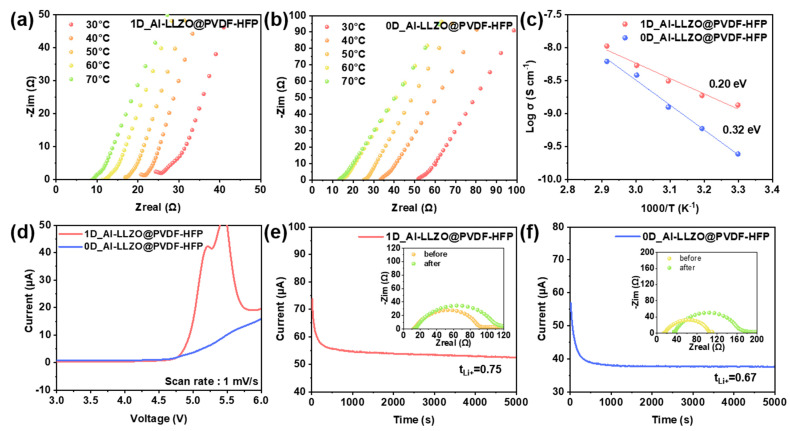
EC properties of 0D_Al-LLZO@PVDF-HFP and 1D_Al-LLZO@PVDF-HFP composite-based solid electrolytes. (**a**) Electrochemical impedance spectroscopy (EIS) spectra of 0D_Al-LLZO@PVDF-HFP and (**b**) 1D_Al-LLZO@PVDF-HFP; (**c**) Arrhenius plots of ionic conductivity for 0D_Al-LLZO@PVDF-HFP and 1D_Al-LLZO@PVDF-HFP; (**d**) comparison of the LSV curves of 0D_Al-LLZO@PVDF-HFP and 1D_Al-LLZO@PVDF-HFP for the measurement of EC stability; DC polarization curve based on Li symmetric cell for (**e**) 0D_Al-LLZO@PVDF-HFP and (**f**) 1D_Al-LLZO@PVDF-HFP.

As displayed in Figure 3, the ionic conductivity (Figure 3a–c), electrochemical window (Figure 3d), polarization curves, and the Nyquist plot (Figure 3e,f) were obtained for the Al-LLZO@PVDF-HFP composite-based solid electrolytes (0D_Al-LLZO@PVDF-HFP and 1D_Al-LLZO@PVDF-HFP) for comparative analyses.

As compared in the Nyquist plots (Figure 3a,b, Figures S4 and S5), 0D_Al-LLZO@PVDF-HFP exhibits ionic conductivities of 6.66 × 10^−5^ S/cm at 30 °C and 2.20 × 10^−4^ S/cm at 60 °C, while 1D_Al-LLZO@PVDF-HFP displays enhanced ionic conductivities of 1.40 × 10^−4^ S/cm at 30 °C and 3.42 × 10^−4^ S/cm at 60 °C, higher than that of 0D_Al-LLZO@PVDF-HFP and PVDF-HFP (5.88 × 10^−^^5^ S/cm at 30 °C). Such higher conductivity of 1D_Al-LLZO@PVDF-HFP is attributed to the unique nanowire-based 1D structure of the Al-LLZO in the polymer matrix, acting as a major ionic conductor. Figure S5 shows the Nyquist plot measured at 0 °C and −20 °C, respectively, used to measure the ionic conductivity (σ) of electrolytes (PVDF-HFP, 0D_Al-LLZO@PVDF-HFP, and 1D_Al-LLZO@PVDF-HFP). As summarized in Table S1, the *R*_b_ values at 0 °C were 93 Ω, 57 Ω, and 45 Ω for PVDF-HFP, 0D_Al-LLZO@PVDF-HFP, and 1D_Al-LLZO@PVDF-HFP, respectively, corresponding to ionic conductivities of 3.79 × 10^−5^ S/cm, 6.19 × 10^−5^ S/cm, and 7.84 × 10^−5^ S/cm. In addition, at the decreased temperature of −20 °C, *R*_b_ increased significantly to 340 Ω (PVDF-HFP), 260 Ω (0D_Al-LLZO@PVDF-HFP), and 156 Ω (1D_Al-LLZO@PVDF-HFP), indicating the reduced ionic conductivities of 1.04 × 10^−5^ S/cm 340 Ω (PVDF-HFP), 1.36 × 10^−5^ S/cm (0D_Al-LLZO@PVDF-HFP), and 2.26 × 10^−5^ S/cm (1D_Al-LLZO@PVDF-HFP), respectively. These results indicate the EC performance and electrical properties of the composite electrolytes depending on the temperature and composite structure by showing increased resistance and reduced ionic conductivity at lower temperatures and polymer or 0D structured composite electrolytes. Notably, 1D_Al-LLZO@PVDF-HFP exhibited the highest ionic conductivity among all electrolytes at all temperature ranges (0 °C and −20 °C), implying facilitated lithium-ion transport in the 1D nanowire structured composite electrolyte even in the low-temperature condition. As the activation energy required for lithium-ion transport of solid electrolytes determines the diffusivity of ions, we compared the activation energy of electrolytes by drawing an Arrhenius plot obtained at various temperatures [74,75]. As shown in Figure 3c, 1D_Al-LLZO@PVDF-HFP exhibited a lower activation energy (0.20 eV) compared to 0D_Al-LLZO@PVDF-HFP (0.32 eV). This enhanced ionic conductivity and reduced activation energy can be attributed to the unique structural characteristics of 1D_Al-LLZO@PVDF-HFP, which facilitate Li^+^ transport and enhance lithium dissociation [77,78]. As displayed in the LSV curve for PVDF-HFP, 0D_Al-LLZO@PVDF-HFP, and 1D_Al-LLZO@PVDF-HFP (Figure S6 and Figure 3d) analyzed using a Li|SS cell to compare their electrochemical stability (electrochemical window), PVDF-HFP exhibits a voltage window of 4.50 V vs. Li/Li+. 1D_Al-LLZO@PVDF-HFP exhibited an expanded EC window (4.75 V) wider than that of 0D_Al-LLZO@PVDF-HFP (4.65 V), indicating significantly improved EC stability and sustainability of 1D_Al-LLZO@PVDF-HFP compared with that of controls (0D_Al-LLZO@PVDF-HFP and PVDF-HFP) at various voltage ranges (Figure S6). Figure 3e,f display the DC polarization curves and the Nyquist plot of 0D_Al-LLZO@PVDF-HFP and 1D_Al-LLZO@PVDF-HFP measured with a Li symmetric cell in the initial and steady state. Based on the EIS analyses, Li^+^ transference numbers (t_Li+_) for 0D_Al-LLZO@PVDF-HFP and 1D_Al-LLZO@PVDF-HFP were calculated using Equation (3) for the evaluation of Li^+^ transfer ability in solid electrolytes. 1D_Al-LLZO@PVDF-HFP shows larger t_Li+_ (0.75) compared to 0D_Al-LLZO@PVDF-HFP (0.67), suggesting enhanced Li^+^ transfer ability (enhanced Li^+^ transport) of the 1D-based composite electrolyte. Consistently, as shown in Table 2, 1D_Al-LLZO@PVDF-HFP exhibited enhanced EC properties compared to that of 0D_Al-LLZO@PVDF-HFP owing to a stabilized electrolyte/electrode interface and facilitated Li^+^ transport channels due to the 1D Al-LLZO filler network in the polymer matrix.

Overall, the proposed 1D_Al-LLZO@PVDF-HFP composite electrolyte demonstrated enhanced ionic conductivity of 1.40 × 10^−4^ S/cm, a high lithium-ion transference number (0.75) at 30 °C, and a wider EC stability window (4.75 V) compared to the controls (PVDF-HFP and 0D_Al-LLZO@PVDF-HFP). The excellent EC properties of 1D_Al-LLZO@PVDF-HFP result in the EC performance exceeding that of previously reported polymer–ceramic composite electrolytes (~1.0 × 10^−4^ S/cm ionic conductivity, t_Li+_ > 0.7) [79,80]. Such a good EC performance of 1D_Al-LLZO@PVDF-HFP is attributed to the continuous Li^+^ ion transport channel through ceramic fillers, reduced activation energy (0.20 eV), and suppressed electrode/electrolyte interfacial resistance due to 1D Al-LLZO nanowire fillers within the polymer [81].

**Figure 4:**
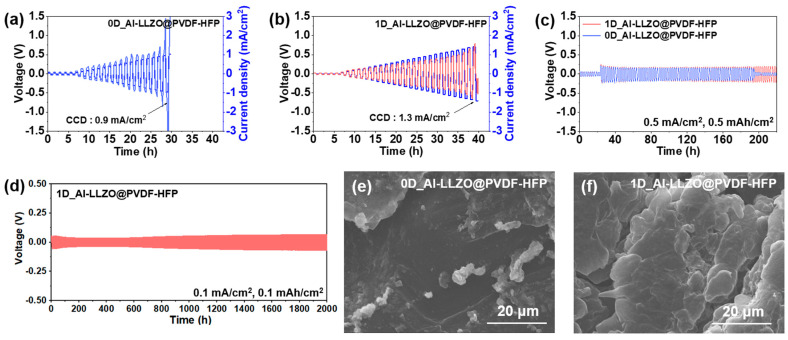
Galvanostatic cycling tests of the Li|0D_Al-LLZO@PVDF-HFP|Li and Li|1D_Al-LLZO@PVDF-HFP|Li symmetric cells with current density from 0.1 to 2.5 mA. Critical current density (CCD) test of (**a**) Li|0D_Al-LLZO@PVDF-HFP|Li and (**b**) Li|1D_Al-LLZO@PVDF-HFP|Li at 30 °C (blue line: current profile, red line: voltage profile). (**c**) Galvanostatic cycling tests of Li symmetric cells at a current density of 0.5 mA/cm^2^. (**d**) Long-term cycling performance of Li symmetric cells at a current density of 0.1 mA/cm^2^. SEM images of the lithium metal anode surfaces in Li symmetric cells after 220 h of cycling at 0.5 mA/cm^2^ assembled with (**e**) 0D_Al-LLZO@PVDF-HFP and (**f**) 1D_Al-LLZO@PVDF-HFP.

As shown in Figure 4, the interfacial stability of the composite electrolyte (1D_Al-LLZO@PVDF-HFP) was evaluated through stripping/plating tests (Figure 4a,b and Figure S7), galvanostatic charge/discharges (Figure 4c), and the cycling test (Figure 4d). Additionally, the surface morphology of lithium metal adopting 1D_Al-LLZO@PVDF-HFP and 0D_Al-LLZO@PVDF-HFP was examined after long-term cycling.

To confirm the Li dendrite suppression ability of the electrolyte in the lithium metal batteries, a critical current density (CCD) test was performed for solid electrolytes (PVDF-HFP, 0D_Al-LLZO@PVDF-HFP, and 1D_Al-LLZO@PVDF-HFP) using Li symmetric cells (Figure 4a,b and Figure S7a). CCD is a critical metric for evaluating the ability of solid electrolytes to suppress lithium dendrite formation, allowing for the assessment of the electrolyte’s electrochemical stability [82,83]. That is, the CCD of Li symmetric cells adopting various electrolytes was evaluated to compare the CCD values depending on the electrolytes. In comparative Li symmetric cell tests adopting various electrolytes, 1D_Al-LLZO@PVDF-HFP showed an increased CCD of 1.3 mA/cm^2^ (Figure 4b) higher than that of PVDF-HFP (0.3 mA/cm^2^ (Figure S7a)) and 0D_Al-LLZO@PVDF-HFP (0.9 mA/cm^2^, Figure 4a), suggesting the suppressed growth of lithium or Li dendrite by Al-LLZO(1D) networks and the advantageous feature of 1D_Al-LLZO@PVDF-HFP in the application of high current density batteries. Figure 4c and Figure S7b compare the cycle stability of Li symmetric cell adopting various electrolytes (PVDF-HFP, 0D_Al-LLZO@PVDF-HFP, and 1D_Al-LLZO@PVDF-HFP). The experiment was conducted under a current density of 0.5 mA/cm^2^ and a capacity of 0.5 mAh/cm^2^ in the symmetric cells. The PVDF-HFP-based Li symmetric cell shows rapidly increased overpotential after cycling for 100 h, followed by a short circuit. In contrast, organic/inorganic composite electrolytes of 0D_Al-LLZO@PVDF-HFP and 1D_Al-LLZO@PVDF-HFP containing Al-LLZO filer exhibited reduced overpotentials. Among all the electrolytes, 1D_Al-LLZO@PVDF-HFP exhibited the most stable voltage profile after long cycles, superior to that of 0D_Al-LLZO@PVDF-HFP, which showed a short circuit after 190 h cycling. As displayed in Figure 4d, the long-term stability of 1D_Al-LLZO@PVDF-HFP in the Li symmetric cell was tested at a current density of 0.1 mA/cm^2^ and one-hour intervals. The Li| 1D_Al-LLZO@PVDF-HFP|Li cell demonstrated stable cycling performance for 2000 h. The enhanced EC performance of 1D_Al-LLZO@PVDF-HFP was attributed to the unique composite structure of Al-LLZO(1D) dispersed in the PVDF-HFP matrix, contributing to stable Li^+^ transport during lithium stripping/plating, which ensured long cycle interfacial stability and suppressed dendrite growth [84]. Figure 4e,f and Figure S8 present the top-view and cross-sectional SEM images of lithium surfaces under various electrolytes (PVDF-HFP, 0D_Al-LLZO@PVDF-HFP, and 1D_Al-LLZO@PVDF-HFP) after 200 h of cycling at 0.5 mA/cm^2^. As displayed in Figure S8a, an irregular deposition with noticeable cracks and non-uniform surfaces was observed for the after-cycled lithium under PVDF-HFP. In comparison, SEM images for the lithium under 0D_Al-LLZO@PVDF-HFP (Figure 4e) demonstrate a more uniform surface and reduced cracks or voids compared to that of lithium under PVDF-HFP. Very differently, as shown in the SEM image for the lithium under 1D_Al-LLZO@PVDF-HFP (Figure 4f), a significantly flat and uniform surface was observed, outperforming that of the lithium under control electrolytes (0D_Al-LLZO@PVDF-HFP and PVDF-HFP). Additionally, as displayed in the cross-sectional images (Figure S8d–f), an uneven lithium layer was deposited under PVDF-HFP (Figure S8d), while localized irregular lithium layers were observed under the 0D_Al-LLZO@PVDF-HFP (Figure S8e). In contrast, a uniform and smooth lithium layer was formed under 1D_Al-LLZO@PVDF-HFP (Figure S8f). This indicates unstable lithium deposition when using PVDF-HFP and 0D_Al-LLZO@PVDF-HFP. Moreover, these cross-sectional images are in good agreement with the top-view observations (Figure S8a–c).

Overall, our EC results confirm the excellent EC properties of the 1D Al-LLZO nanowire-based composite electrolyte, including significantly higher ionic conductivity (1.40 × 10^−4^ S/cm), lower interfacial resistance (25 Ω), and a higher lithium transference number (0.75) superior to that of conventional 0D nanoparticle-based composite electrolytes. These findings clearly highlight the structural and electrochemical advantages of 1D Al-LLZO nanowires in hybrid solid electrolytes by demonstrating effective immobilized TFSI^−^ anions within the composite due to its Lewis acid characteristics and enhanced lithium transference number (tLi^+^); such suppressed excessive Li^+^ concentration gradients in LMB employing the 1D Al-LLZO nanowire-based composite electrolyte could effectively mitigate lithium dendrite formation, contributing to improved overall electrochemical stability [85].

**Figure 5:**
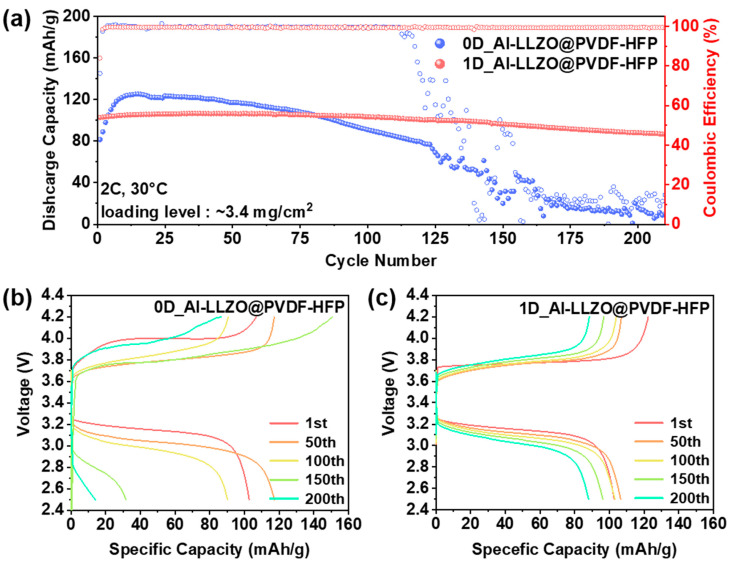
(**a**) Cycling performance of the Li|0D_Al-LLZO@PVDF-HFP|LFP and Li|1D_Al-LLZO@PVDF-HFP|LFP cells at 2C (1C = 170 mA/g). Solid dots indicate the voltage profile, and hollow dots indicate the Coulombic efficiency (%) corresponding to the right y-axis. Charge/discharge voltage profile of LFP in the cell employing the electrolyte of (**b**) 0D_Al-LLZO@PVDF-HFP and (**c**) 1D_Al-LLZO@PVDF-HFP.

Figure 5 compares the EC performance of the full cell (Li|electrolyte|LFP) by adopting the composite electrolyte of 0D and 1D Al-LLZO@PVDF-HFP under 2.5–4.2 V and at 2C (1C = 170 mA/g). As displayed in the comparative cyclic performance (Figure 5a), the 1D_Al-LLZO@PVDF-HFP-based cell (Li|1D_Al-LLZO@PVDF-HFP|LFP) exhibited stable performance over 200 cycles, while the 0D_Al-LLZO@PVDF-HFP-based cell experienced a short circuit after 125 cycles. In addition, the 1D_Al-LLZO@PVDF-HFP electrolyte-based cell demonstrated an excellent capacity for retention (C/C_0_: 85.7% after 200 cycles), with a high coulombic efficiency of 99.4%. Such EC performances were further compared with voltage profiles of LFP with 0D and 1D-based composite electrolytes obtained at various cycles (Figure 5b,c). As displayed in the voltage profiles for the LFP cathode adopting 0D_Al-LLZO@PVDF-HFP (Figure 5b) and 1D_Al-LLZO@PVDF-HFP (Figure 5c) as an electrolyte, 1D_Al-LLZO@PVDF-HFP showed a higher initial specific capacity of 102.7 mAh/g than that of 0D_Al-LLZO@PVDF-HFP (81.5 mAh/g). Specifically, the LFP cathode with 0D_Al-LLZO@PVDF-HFP exhibited an unstable voltage profile with a drastic capacity decay and increased voltage polarization at elongated cycles, while the cathode with 1D_Al-LLZO@PVDF-HFP demonstrated a stable voltage profile and stable plateaus after 200 cycles without side reactions or drastic capacity decay. It is important to note that 0D_Al-LLZO@PVDF-HFP exhibited a higher capacity in the early cycles due to facilitated lithium-ion transport, facile lithium salt dissociation, and larger active area due to nanoparticles in the composite. However, the agglomeration tendency of nanoparticles in the polymer induced non-uniform defects and interfaces within the composite, leading to capacity degradation and short circuits due to promoted lithium dendrites over cycles [65]. In contrast, 1D_Al-LLZO@PVDF-HFP with a continuous 1D network structure can facilitate lithium-ion transport due to a paved 1D ion transport channel, promoting uniform lithium stripping and plating at elongated cycles. Specifically, the minimized structural defects and sustained ion-conducting channels by nanowires can effectively suppress Li dendrite growth, ensuring long-term stability [82]. Additionally, nanowire fillers with strong Lewis acid–base interaction with lithium-ion vacancies further enhance lithium-ion dissociation and transport within the composite [81]. Consistent with comparative CCD analyses of the electrolytes (Figure 4), the nanowire filler-based composite electrolyte (1D_Al-LLZO@PVDF-HFP) demonstrates superior electrochemical performance (long-term cycle stability) compared to 0D_Al-LLZO@PVDF-HFP, effectively facilitating channel formation within the composite and minimizing structural defects [86].

**Figure 6:**
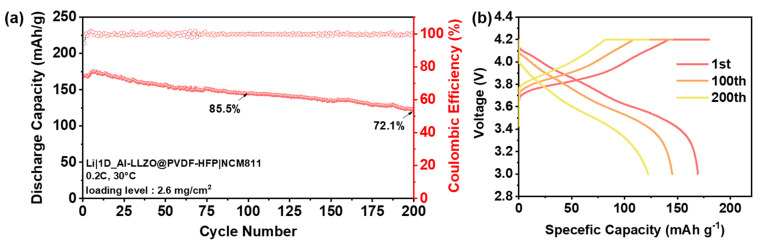
(**a**) Cycling performance of the Li|1D_Al-LLZO@PVDF-HFP|NCM811 cell at 0.2C (1C = 215 mAh/g, loading level: 2.6 mg/cm^2^). Solid red dots represent the voltage profile, and hollow red dots indicate the Coulombic efficiency (%) corresponding to the right y-axis. (**b**) Voltage profile of 1D_Al-LLZO@PVDF-HFP at initial (1st) and subsequent cycles (100th, 200th cycle).

Figure 6 shows the EC performance of the full cell by employing 1D_Al-LLZO@PVDF-HFP as an electrolyte and lithium iron phosphate (LFP) as a cathode conducted at the range of 3.0–4.2 V under a current density of 0.2 C (1C = 215 mA/g). As shown in Figure 6a, the cell shows an initial specific capacity of 169.4 mAh/g, good cyclic capacity retention (C/C_0_: 85.5% after 100 cycles; 72.1% after 200 cycles), and coulombic efficiencies (100%) at elongated cycles. Figure 6b shows the voltage profiles for the LFP cathode obtained at the 1st, 100th, and 200th cycle. During the 1st and 100th cycles, a reversible charge/discharge could be obtained without a drastic polarization but with gradual capacity decay after 200 cycles. Despite the deteriorated behavior of the cathode at extended cycles due to the crystal structure changes of the cathode material [87], our cell still shows high-capacity retention over 120 mAh/g after elongated cycles (>200 cycles), suggesting the stable EC operability of 1D_Al-LLZO@PVDF-HFP as an electrolyte. It is important to note that, compared with the EC performance in previously reported studies (Table S2), our 1D_Al-LLZO@PVDF-HFP-based EC system exhibits superior performance, with a high lithium-ion transference number of 0.75, a high reversible capacity (LFP, 102.7 mAh/g at 2C), and excellent Coulombic efficiency (~99.4%) compared to that of references.

Overall, as demonstrated by the comparative EC performance, the 1D_Al-LLZO@PVDF-HFP composite solid electrolytes exhibit superior performance to that of 0D_Al-LLZO@PVDF-HFP. It is important to note that, despite similar components and composition to the composite (PVDF-HFP/LiTFSI/SN + Al-LLZO), 1D_Al-LLZO@PVDF-HFP displays enhanced lithium salt dissociation, reduced polymer crystallinity, facilitated ion transport, and suppressed lithium dendrite growth, highlighting the importance of the composite structure in the performance.

**Figure 7:**
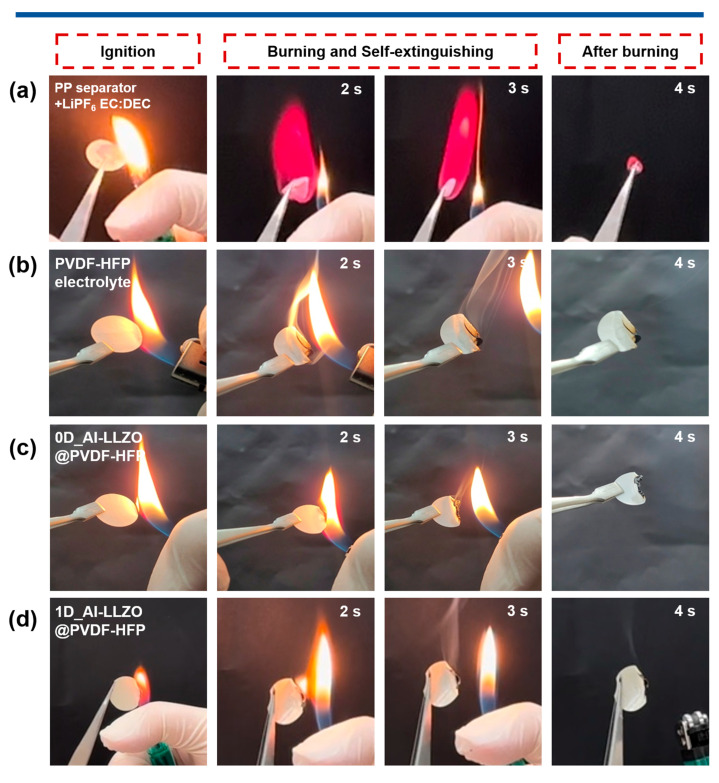
Comparative flammability test for (**a**) liquid electrolyte (PP separator + 1M LiPF_6_ EC:DEC), (**b**) 1D_Al-LLZO@PVDF-HFP composite electrolyte, (**c**) 0D_Al-LLZO@PVDF-HFP composite electrolyte, and (**d**) 1D_Al-LLZO@PVDF-HFP composite-based solid electrolyte.

As shown in Figure 7, the flammability test was conducted to evaluate the fire safety of electrochemical cells based on various electrolytes (liquid, PVDF-HFP, 0D, and 1D_Al-LLZO@PVDF-HFP). Regarding the flammability test of the liquid electrolyte, the polypropylene (PP) separator was sufficiently soaked in 50 μL of 1 M LiPF_6_ EC:DEC (1:1 vol%) and used as a sample for the test. In addition, solid electrolytes, including PVDF-HFP and Al-LLZO@PVDF-HFP (0D and 1D), were prepared at an identical size of PP separator to be used for the fire safety test without additional treatment. Both samples were ignited with fire and burned in ambient conditions.

Figure 7a illustrates the flammability of a PP separator impregnated with liquid electrolyte (LiPF6 EC:DEC), which rapidly catches fire upon exposure to a flame. In contrast, as shown in Figure 7b, PVDF-HFP (PVDF-HFP/LiTFSI/SN) initially ignited, but the flame was extinguished over time with partial polymer charring. Figure 7c presents the fire test results for 0D_Al-LLZO@PVDF-HFP composed of Al-LLZO(0D) and PVDF-HFP. In the fire test, partial polymer charring was observed; however, no continuous combustion occurred for 0D_Al-LLZO@PVDF-HFP. As shown in Figure 7d, a similar flammability result with 0D_Al-LLZO@PVDF-HFP was observed for the 1D_Al-LLZO@PVDF-HFP containing Al-LLZO(1D). These findings provide important insights into the enhanced fire resistance and battery safety of electrolytes containing inorganic fillers.

Overall, in the flammability test, the PP separator impregnated with liquid electrolyte (LiPF_6_ EC:DEC) exhibited high flammability of rapid ignition and sustained combustion when exposed to a flame. In contrast, PVDF-HFP (PVDF-HFP/LiTFSI/SN) displayed self-extinguish over time after the initial fire flame with partial polymer charring and superior fire safety to that of the liquid electrolyte. The 0D and 1D_Al-LLZO@PVDF-HFP composite electrolyte showed the best fire resistance of partial polymer charring without sustained combustion. These results highlight the importance of incorporating inorganic fillers in the composite electrolyte to achieve enhanced fire safety of batteries.

## 4. Conclusions

In this study, we systematically examined the effect of the structure of a composite electrolyte on the EC performance and fire safety by comparing EC properties and fire safety of Al-LLZO(0D) and Al-LLZO(1D) dispersed in a PVDF-HFP/LiTFSI/SN-based polymer matrix. Here, we summarized the comparative results (material property, EC property, and fire safety) of a composite electrolyte as a function of their morphological structure. In addition, the structural novelties and uniqueness of the 1D Al-LLZO nanowire-based composite electrolyte are summarized by highlighting the structural uniqueness enabling continuous ion conduction pathways and promoted lithium salt dissociation.

First, an analysis of the crystal structure (XRD) and morphological structure (SEM) confirmed the regulated synthesis of 0D and 1D Al-LLZO@PVDF-HFP composite electrolyte. Additionally, FT-IR and Raman spectroscopy were used to compare the effect of the morphological differences of Al-LLZO dispersed in the PVDF-HFP-based electrolyte on lithium salt dissociation. The results indicate that the 1D structure promotes lithium salt dissociation and enhances ion mobility. 0D Al-LLZO in PVDF-HFP can improve lithium salt dissociation and enhance initial lithium mobility but tend to aggregate. In contrast, 1D Al-LLZO in PVDF-HFP can sustain ion conduction over extended cycles with the retention of stable ionic transport networks.

Second, in the comparative EC characterization (symmetric cells test), the 1D_Al-LLZO@PVDF-HFP composite with a continuous ion transfer ability and reduced interfacial resistance exhibited enhanced ionic conductivity (1.40 × 10^−4^ S/cm) and electrochemical stability (higher CCD (1.3 mA/cm^2^) and stable cycling performance (>2000 h)) at 4.75 V, superior to that of the 0D_Al-LLZO@PVDF-HFP composite (earlier polarization and lower CCD (0.9 mA/cm^2^)) under identical condition due to localized ion conduction paths and interfacial resistance created by nanoparticle fillers. In full-cell tests, the 1D_Al-LLZO@PVDF-HFP composite maintained stable transport networks, ensuring long-term retention (85.7% over 200 cycles), superior to that of the 1D_Al-LLZO@PVDF-HFP composite suffering from aggregation-induced capacity fading.

Third, in the fire safety test, both 1D and 0D _Al-LLZO@PVDF-HFP exhibited similar fire safety (flammability), suggesting the morphological structure-independent flammability of the composite electrolyte. Nevertheless, the inorganic filler in solid electrolytes plays an important role as a flame-retardant material to enhance the fire safety and thermal stability of the batteries.

As demonstrated in this study, we systematically analyzed the impact of the inorganic filler morphology on lithium-ion transport, lithium salt dissociation, and electrochemical stability to highlight the unique structure of the 1D nanowire network composite electrolyte (1D Al-LLZO@PVDF-HFP). The 1D Al-LLZO@PVDF-HFP composite electrolyte containing a continuous percolation network of 1D nanowires exhibits enhanced ionic transport (ionic conductivity) and superior electrochemical performance and fire safety compared to PVDF-HFP and 0D Al-LLZO@PVDF-HFP composite electrolytes by effectively utilizing the network structure of 1D ceramic ionic conductors in the composite electrolyte.

It should be followed as future work to serve our composite electrolyte as a promising alternative for next-generation solid-state lithium-metal batteries (SSE-LMBs) with enhanced performance and safety. A forthcoming study includes (1) the novel design of ceramic fillers to improve ionic conductivity, mechanical strength, and thermal stability; (2) the incorporation of new bonding between the polymer matrix and the filler to reduce the interfacial resistance; and (3) improvement in the long-term stability and durability of electrolytes.

## Figures and Tables

**Table 1:** Sample names and their corresponding full descriptions used in this study.

Sample Name	Full Name
PVDF-HFP	PVDF-HFP/LiTFSI/SN
0D_Al-LLZO@PVDF-HFP	Al-LLZO(0D)+PVDF-HFP/LiTFSI/SN
1D_Al-LLZO@PVDF-HFP	Al-LLZO(1D)+PVDF-HFP/LiTFSI/SN

**Table 2:** Ionic conductivity, activation energy, transference number, and electrochemical window of 0D_Al-LLZO@PVDF-HFP and 1D_Al-LLZO@PVDF-HFP.

Sample	σ (S/cm)	E_a_ (eV)	t_Li+_	Electrochemical Window (V)
0D_Al-LLZO @PVDF-HFP	0.67 × 10^−4^ (30 °C)	0.32	0.67	4.65
1D_Al-LLZO @PVDF-HFP	1.40 × 10^−4^ (30 °C)	0.20	0.78	4.75

## Data Availability

The original contributions presented in the study are included in the article/supplementary material, further inquiries can be directed to the corresponding author.

## Supplementary Materials

The following supporting information can be downloaded at: https://www.mdpi.com/article/10.3390/ma18071536/s1, Figure S1: SEM images of Al-LLZO(1D) (a) as-prepared by electrospinning and (b) after sintering at 750 °C, as well as (c) side view of 1D_Al-LLZO@PVDF-HFP. (d) Elemental mapping images for Al-LLZO nanofiber obtained with SEM-EDS: Zr (blue), La (yellow), Al (green), and O (red); Figure S2: SEM images of the (a) cross-sectional and (b) top-view morphologies of PVDF-HFP; Figure S3: XRD pattern of (a) Al-LLZO, (b) PVDF-HFP, 0D_Al-LLZO@PVDF-HFP, and 1D_Al-LLZO@PVDF-HFP and PVDF-HFP in the two theta ranges of 15° to 25°, as well as (c) SN and LiTFSI; Figure S4: Electrochemical impedance spectroscopy spectra of PVDF-HFP measured at 30 °C; Figure S5: Nyquist plots of PVDF-HFP, 0D_Al-LLZO@PVDF-HFP, and 1D_Al-LLZO@PVDF-HFP electrolytes measured at low temperatures at (a) 0 °C and (b) −20 °C; Figure S6: Linear sweep voltammetry curve of PVDF-HFP measured at 30 °C; Figure S7: Galvanostatic cycling tests of the Li|PVDF-HFP|Li symmetric cell with current density from 0.1 to 2.5 mA. (b) Galvanostatic cycling tests during Li symmetric cell at a current density of 0.5 mA/cm^2^; Figure S8: SEM images of lithium surfaces after 200 h of cycling at 0.5 mA/cm2 with PVDF-HFP, 0D_Al-LLZO@PVDF-HFP, and 1D_Al-LLZO@PVDF-HFP: (a) Top-view SEM images of lithium metal surfaces using (a) PVDF-HFP, (b) 0D_Al-LLZO@PVDF-HFP, and (c) 1D_Al-LLZO@PVDF-HFP. Cross-sectional SEM images of lithium metal showing side views using (d) PVDF-HFP, (e) 0D_Al-LLZO@PVDF-HFP, and (f) 1D_Al-LLZO@PVDF-HFP; Table S1: R_b_ and ionic conductivity of PVDF-HFP, 0D_Al-LLZO@PVDF-HFP, and 1D_Al-LLZO@PVDF-HFP electrolytes measured at 0 °C and −20 °C; Table S2: Comparative EC performance. References [88,89,90,91] are cited in the supplementary materials.
