# Supplementary material for: The Structural Effect of a Composite Solid Electrolyte on Electrochemical Performance and Fire Safety
Source: Materials (Basel). 2025 Mar 28;18(7):1536. doi: 10.3390/ma18071536 (PMC11989986; doi:10.3390/ma18071536)
Supplement: Supplementary file 1 [file materials-18-01536-s001.zip › materials-3270393-supplementary.pdf]

The Structural Effect of Al-doped LLZO/PVDF-HFP Composite Solid Electrolyte on Electrochemical Performance and Fire Safety

Hwiyun Im[1], Dae Ung Park[1], Yong Jae Lee[1], Junseok Moon[1], Sanglim Lee[1], Tae Min Choi[3], Taek Lee[1], Giwon Lee[1], Jong-Min Oh[2], Weon Ho Shin[2], Sung Gyu Pyo[3], Anusorn Seubsai[4], Hiesang Sohn[1,*]

[1]Department of Chemical Engineering, Kwangwoon University, Seoul 01897, Korea
[2]Department of Electronic Material Engineering, Kwangwoon University, Seoul 01897, Korea
[3] School of Integrative Engineering, Chung-Ang University, Seoul 06974, Korea
[4]Department of Chemical Engineering, Faculty of Engineering, Kasetsart University, Bangkok 10900, Thailand

*Correspondence: hsohn@kw.ac.kr, sonisang@hanmail.net (H. Sohn)

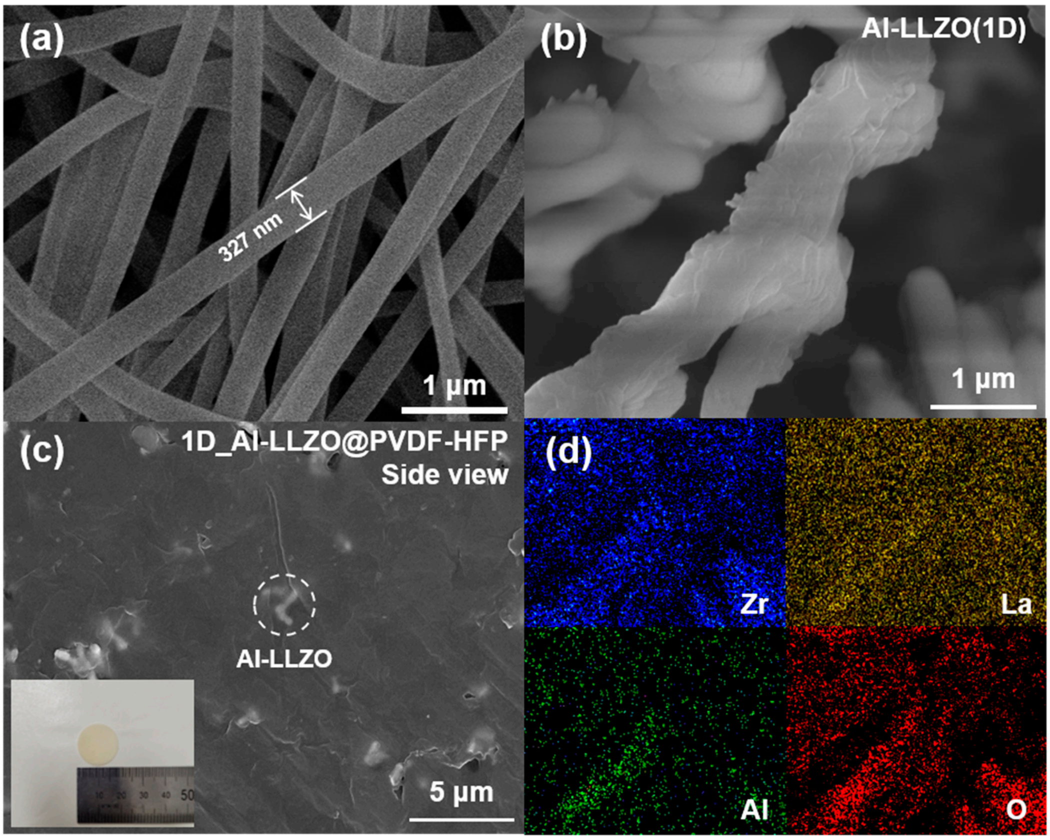

Figure S1. SEM images of Al-LLZO(1D) (a) as-prepared by electrospinning and (b) after sintering at 750°C, (c) side view of 1D_Al-LLZO@PVDF-HFP. (d) Elemental mapping images for Al-LLZO nanofiber obtained with SEM-EDS: Zr (blue), La (yellow), Al (green), O (red).

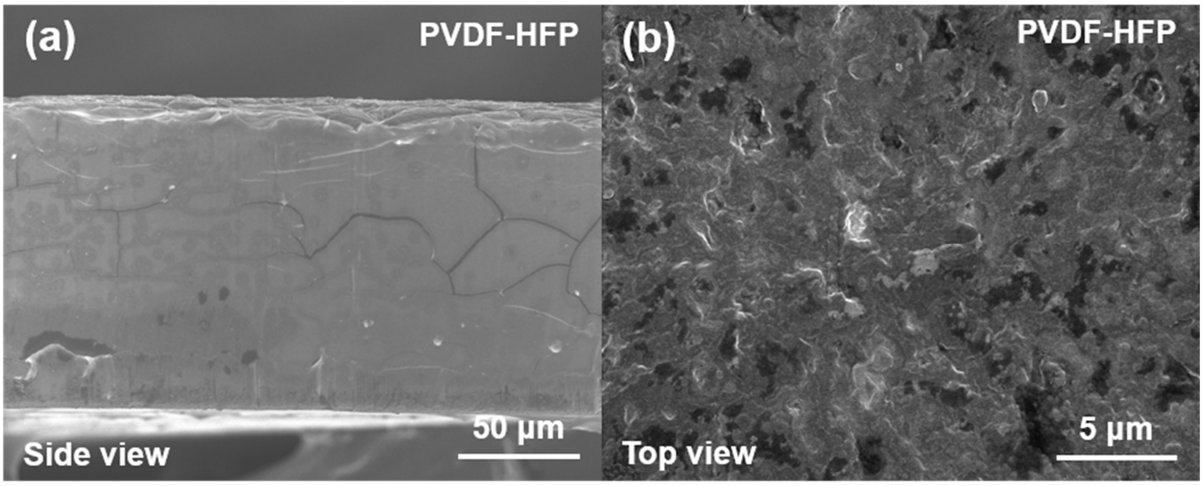

Figure S2. SEM images of the (a) cross-sectional and (b) top-view morphologies of PVDF-HFP.

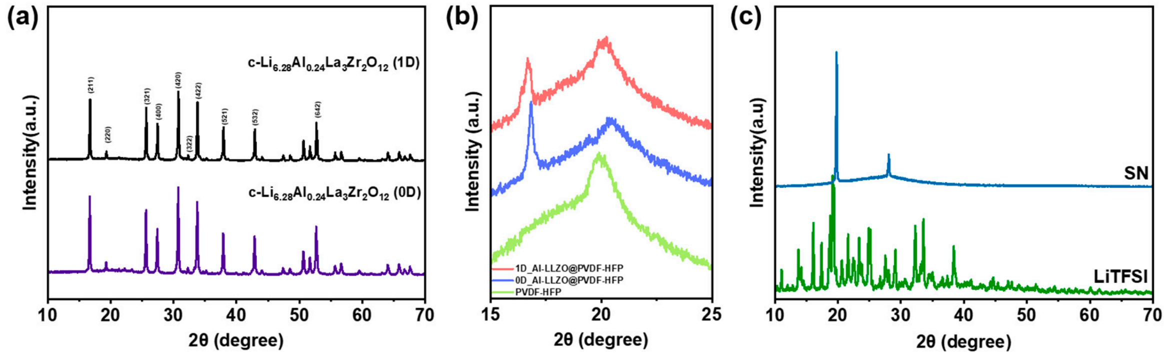

Figure S3. XRD pattern of (a) Al-LLZO, (b) PVDF-HFP, 0D_Al-LLZO@PVDF-HFP and 1D_Al-LLZO@PVDF-HFP in the two theta range of 15° to 25°, and (c) SN and LiTFSI.

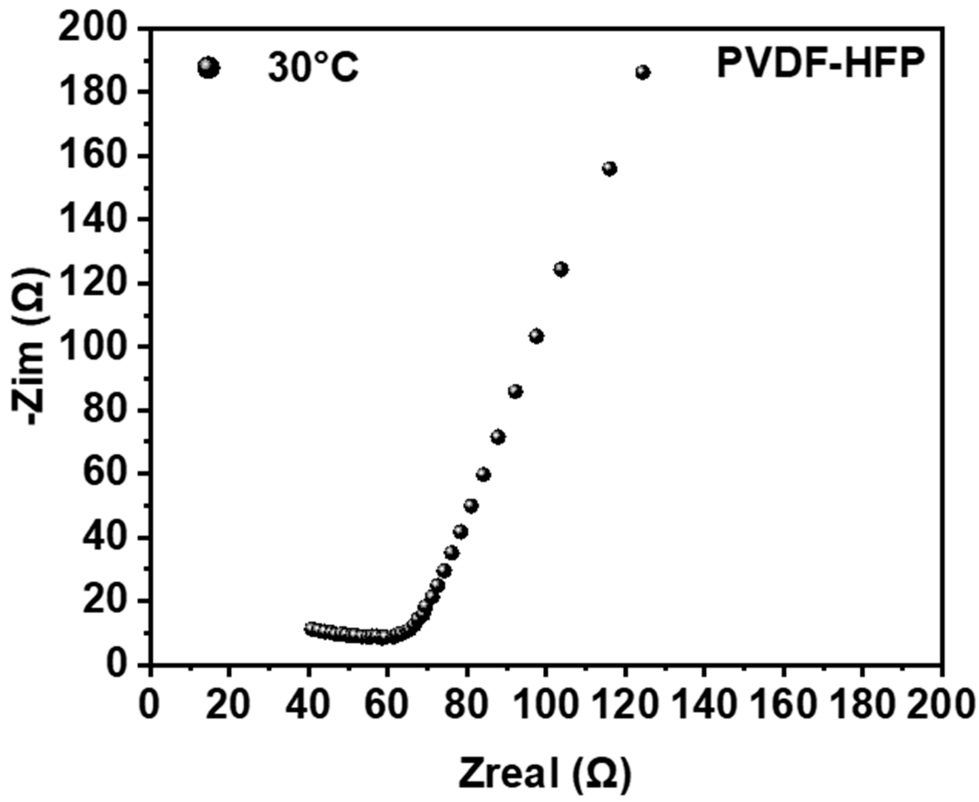

Figure S4. Electrochemical impedance spectroscopy spectra of PVDF-HFP measured at 30°C.

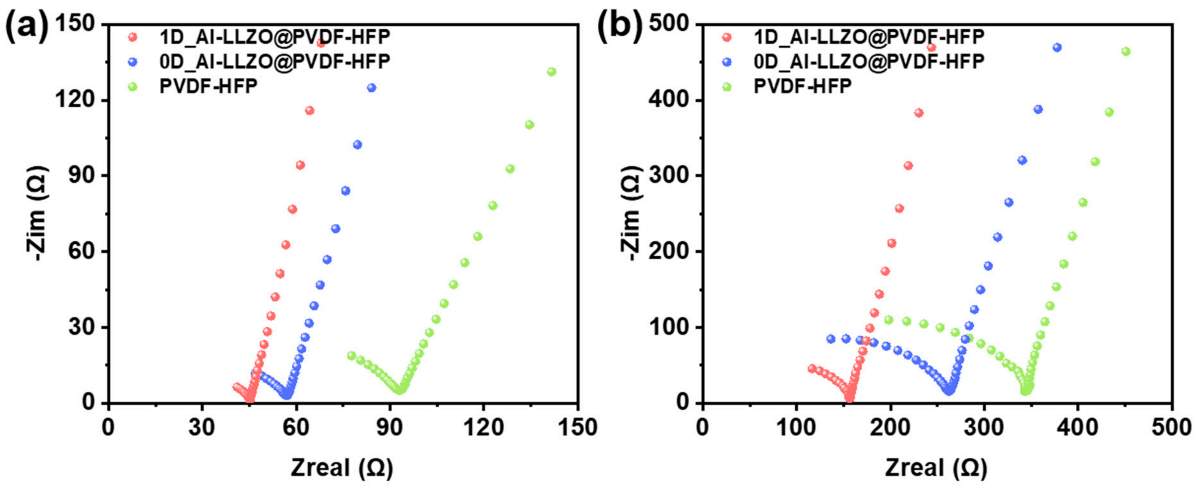

Figure S5. Nyquist plots of PVDF-HFP, 0D_Al-LLZO@PVDF-HFP, and 1D_Al-LLZO@PVDF-HFP electrolytes measured at low temperatures at (a) 0°C and (b) -20°C.

Table S1. R_b and ionic conductivity of PVDF-HFP, 0D_Al-LLZO@PVDF-HFP, and 1D_Al-LLZO@PVDF-HFP electrolytes measured at 0°C and -20°C.

**Sample**	**R_b (Ω) (0°C)**	**R_b (Ω) (-20°C)**	**σ (S/cm) (0°C)**	**σ (S/cm) (-20°C)**
PVDF-HFP	93	340	3.79×10^{-5}	1.04×10^{-5}
0D_Al-LLZO @PVDF-HFP	57	260	6.19×10^{-5}	1.36×10^{-5}
1D_Al-LLZO @PVDF-HFP	45	156	7.84×10^{-5}	2.26×10^{-5}

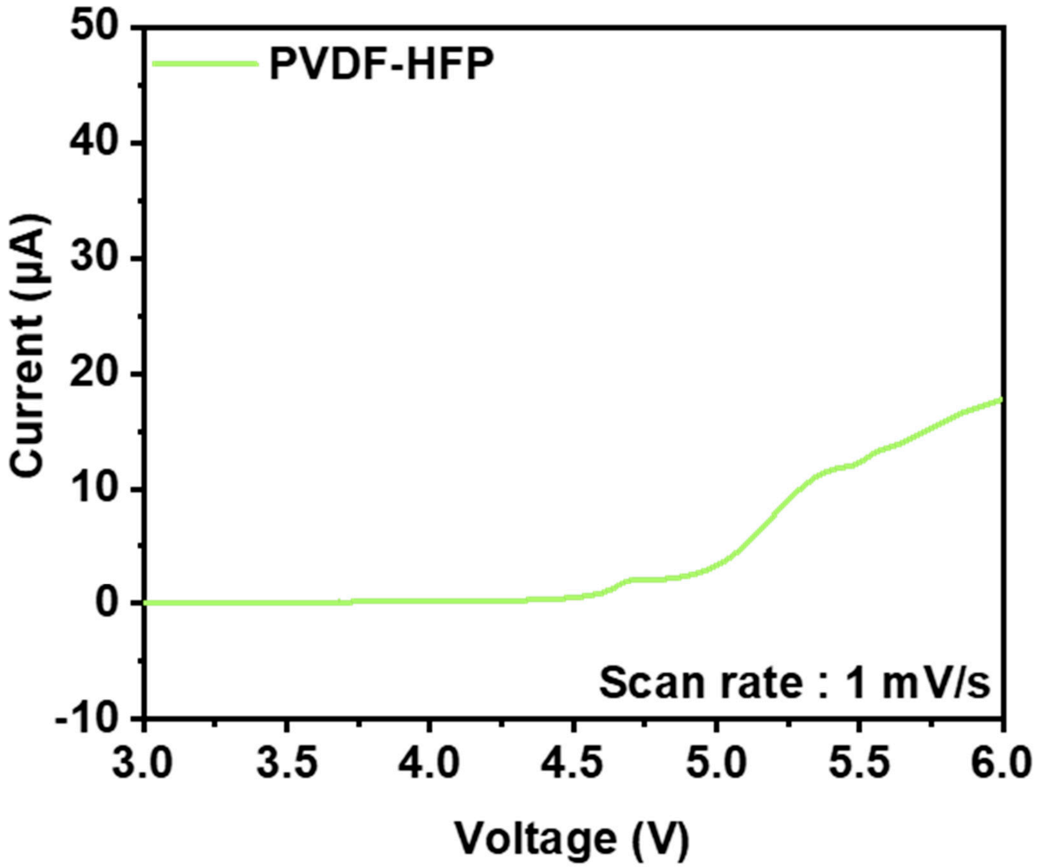

Figure S6. Linear sweep voltammetry curve of PVDF-HFP measured at 30°C.

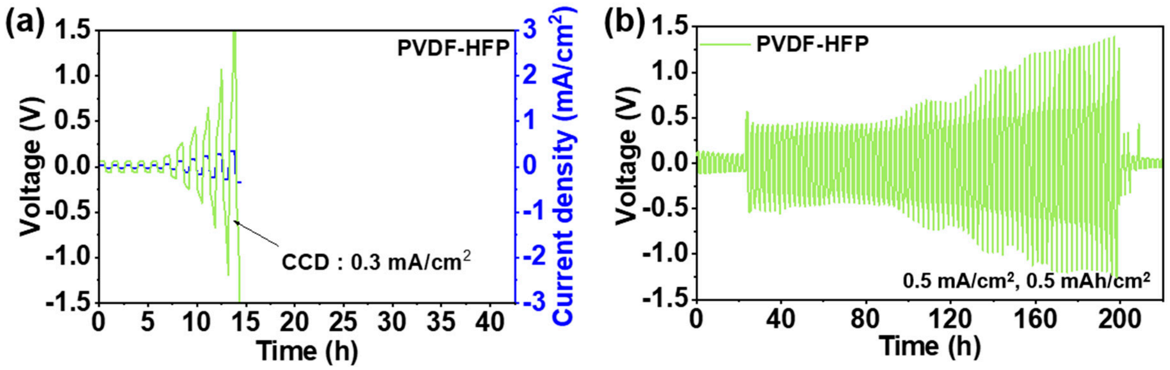

Figure S7. Galvanostatic cycling tests of the Li|PVDF-HFP|Li symmetric cell with current density from 0.1 to 2.5 mA. (b) Galvanostatic cycling tests during Li symmetric cell at a current density of 0.5 mA/cm².

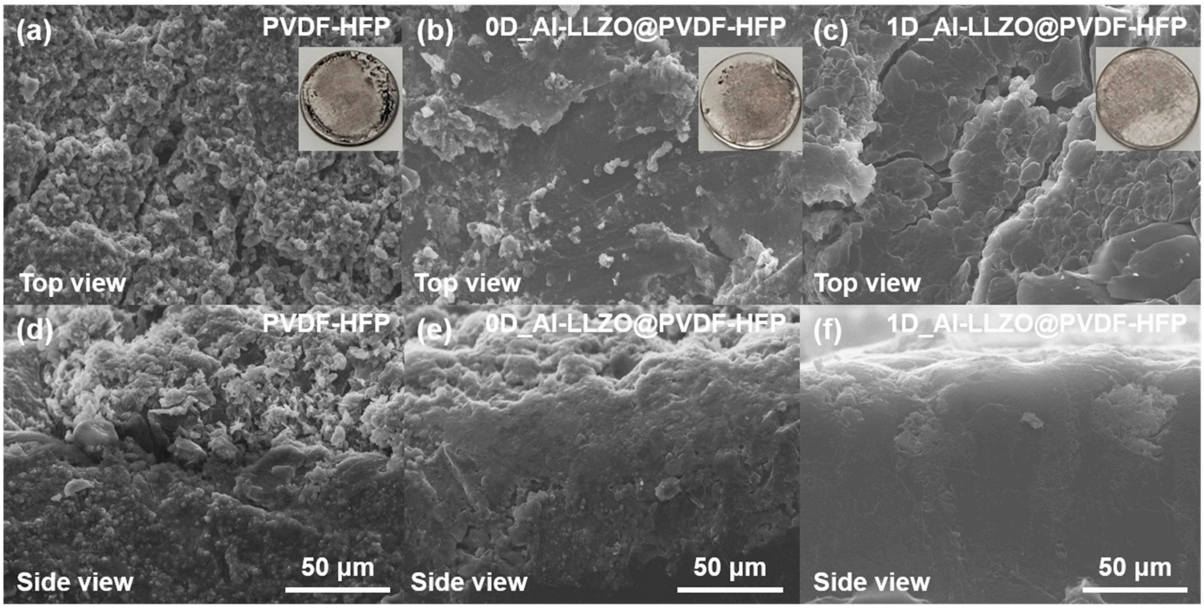

Figure S8. SEM images of lithium surfaces after 200 hours of cycling at 0.5 mA/cm2 with PVDF-HFP , 0D_Al-LLZO@PVDF-HFP, and 1D_Al-LLZO@PVDF-HFP: (a) Top view SEM images of lithium metal surfaces using (a) PVDF-HFP, (b) 0D_Al-LLZO@PVDF-HFP and (c) 1D_Al-LLZO@PVDF-HFP. Cross-sectional SEM images of lithium metal showing side views using (d) PVDF-HFP, (e) 0D_Al-LLZO@PVDF-HFP and (f) 1D_Al-LLZO@PVDF-HFP.

Table S2. Comparative EC performance

Electrolytes	σ [mS/cm^2]	Electrochemical Window [V]	t_{Li+}	LFP Full cell performance [mAh/g]	Capacity Retention	Loading [mg/cm^2]	Ref
PEO/TPU +10wt% LLZO nanowire	0.122 (25℃)	5.6	0.37	144 at 0.5C (60℃) 90 at 2C (60℃)	0.5C 96.1% after 100 cycles	-	[S1]
PEO/LiTFSI +Ca-CeO2 nanotube	0.13 (60℃)	4.5	0.453	121 at 1C (60℃) 100 at 2C (60℃)	1C 76% after 200 cycles	-	[S2]
PVDF-$LiClO_4$+15wt% Al-LLZO nanoparticle	0.15	4.6	-	107 at 0.5C (RT) 97.4 at 1C (RT)	0.5C 80% after 500 cycle	-	[S3]
PEO-LiTFSI+10 wt% LLZTO nanoparticle	0.117 (30℃)	5.0	-	148.6 at 0.2C (55℃) 107.9 at 2C (55℃)	139.1 mAh/g after 100 cycles (0.2C)	2-3	[S4]
PVDF-b-PTFE/LiTFSI+LLZAO/LLTO nanowire	0.138 (25℃)	5.3	0.58	149 at 0.5C (RT) 120 at 1C (RT)	0.5C 85% after 550 cycles	2.5	[S5]
PEO/LiTFSI +LLZO framework	0.137 (30℃)	5.4	0.59	134 at 0.5C (30℃) 85 at 2C (30℃)	-	-	[S6]
**PVDF-HFP/LiTFSI /SN+5wt% 0D Al-LLZO**	**0.067 (30℃) 0.220 (30℃)**	**4.65**	**0.67**	**81.5 at 2C (initial, 30℃)**	**Short after 120 cycles**	**3.4**	**This work**
**PVDF-HFP/LiTFSI /SN+5wt% 1D Al-LLZO**	**0.140 (30℃) 0.342 (60℃)**	**4.75**	**0.75**	**102.7 at 2C (initial, 30℃)**	**2C 85.7% after 200 cycles**		

[S1] Xu, H.; Huang, S.; Qian, J.; Liu, S.; Li, L.; Zhao, X.; Zhang, W. Safe Solid-State PEO/TPU/LLZO Nano Network Polymer Composite Gel Electrolyte for Solid-State Lithium Batteries. *Colloids Surf. A Physicochem. Eng. Asp.* **2022**, *653*, 130040, doi:10.1016/j.colsurfa.2022.130040.

[S2] Chen, H.; Adekoya, D.; Hencz, L.; Ma, J.; Chen, S.; Yan, C.; Zhao, H.; Cui, G.; Zhang, S. Stable Seamless Interfaces and Rapid Ionic Conductivity of Ca–CeO_2/LiTFSI/PEO Composite Electrolyte for High-Rate and High-Voltage All-Solid-State Battery. *Adv. Energy Mater.* **2020**, *10*(21), 2000049, doi:10.1002/aenm.202000049.

[S3] Cheng, B.; Du, P.; Xiao, J.; Zhan, X.; Zhu, L. Improving the Ionic Conductivity and Anode Interface Compatibility of LLZO/PVDF Composite Polymer Electrolytes by Compositional Tuning. *ACS Appl. Mater. Interfaces* **2024**, *16*(24), 31648–31656, doi:10.1021/acsami.4c06803.

[S4] Chen, L.; Li, Y.; Li, S.-P.; Fan, L.-Z.; Nan, C.-W.; Goodenough, J. B. PEO/Garnet Composite Electrolytes for Solid-State Lithium Batteries: From "Ceramic-in-Polymer" to "Polymer-in-Ceramic." *Nano Energy* **2018**, *46*, 176–184, doi:10.1016/j.nanoen.2017.12.037.

[S5] Liu, S.; Zhao, Y.; Li, X.; Yu, J.; Yan, J.; Ding, B. Solid-State Lithium Metal Batteries with Extended Cycling Enabled by Dynamic Adaptive Solid-State Interfaces. *Adv. Mater.* **2021**, *33* (12), 2008084, doi:10.1002/adma.202008084.

[S6] Zhang, H.; An, X.; Lu, Z.; Liu, L.; Cao, H.; Xu, Q.; Liu, H.; Ni, Y. A Three-Dimensional Interconnected $Li_7La_3Zr_2O_{12}$ Framework Composite Solid Electrolyte Utilizing Lignosulfonate/Cellulose Nanofiber Bio-Template for High-Performance Lithium Ion Batteries. *J. Power Sources* **2020**, *477*, 228752, doi:10.1016/j.jpowsour.2020.228752.
